# A European roadmap to a digital epidemiology in public health system

**DOI:** 10.3389/fdgth.2024.1284426

**Published:** 2024-04-19

**Authors:** Mesut Yavuz, Nicolai Savaskan

**Affiliations:** ^1^ Process and Organisational Analysis (IT), YES Automation-Process Management Consulting, Nürnberg, Germany; ^2^ Department of Public Health Neukölln, District Office Neukölln, Berlin, Germany

**Keywords:** digitalization, national public health service, climate change adaptation, online access act, COVID-19, disease outbreaks, one health, pandemic prevention preparedness and response

## 1 Introduction

The year 2020 marks a massive caesura in Germanys municipal public health departments. Never before have public health departments been in the limelight of society and politics (1, 2). However, since almost years into the COVID-19 pandemic, the German public health service still faces the same data management challenges as they dealt with long time before the pandemic. In fact, German public services (ÖGD) and their institutions (Gesundheitsämter, Robert Koch Insitute/RKI) are digitally far beyond the state-of-the-art standard compared to the overall digitalization level of their citizens and enterprises. The organizational and efficiency structures of public services and municipal administration are generally closed systems. Often these systems are outsourced and hosted by companies making institutions heavily depending on single manufactures. Within such dependency face public health institutions often outdated software versions and almost no difficult fall-back solutions in cases of emergencies. This disadvantage is especially overlooked in pandemic preparedness plannings.

Two drivers for digitalization in public service force the process of change: The federal government's efforts need to lawfully implement the European Union digital services act (Germanys Onlinezugangsgesetz) of the European union (3). Secondly, a specific funding act for the digitalization of the Public Health Service (ÖGD-Pakt) was enrolled with up to 800 million Euros. This funding program was aimed to enforce digitalization in the public health sector with 383 public health departments and 16 ministries of health and the national health institutions (4–6). However, the digitization and the interoperability of the underlying infrastructure has not yet turned into success. After the COVID-19 pandemic the question remains what the reasons for the lame introduction of digital solutions are (5).

## 2 Digitalization as a bottleneck in public health service

In 2020, the COVID-19 pandemic caused instantly a massive disruption of health and care services. In particular, the overburdened public health agencies and their digital backlog in Germany was identified as a major bottleneck in health systems (7). The German public health service is a weak institution compared to primary and academic health care institutions in Germany for historical reasons of the murderous health policy during the Nazi regime. However, this does not explain why the technical operation disrupted during the starting pandemic.

### 2.1 Missing consensus on digital public health service

In fact, before the COVID-19 pandemic the public health service was not intended to operate with epidemiological techniques and did not investigate health data. The public health service was rather regarded as another unit of the public administration. In the sense of the Prussian approach of administration, public health service was restricted solely to reporting surveillance data to higher responsibility. In addition, there exist a great misunderstanding and disagreements of the targets and definition of digitization and digital infrastructure in public service till today (8, 9). The federal structure of the public health funding program in Germany brings up different views of what digitalization is mainly good for public health authorities. There does not exist any consent about public health digitalization in Germany. Conversely, the needs and views of community health authorities have neither been registered nor addressed. Thus, fundamental questions such as whether smartphones or whiteboards are already defined as digitalization of public administration employees remains open. What should be the common body of digital infrastructure? How should public health services be defined for digitalization? These issues remain be to addressed. Consequently, major landmarks for public health service digitalization remain unset. Therefore, the goals of public health service digitalization have neither been framed on the community level nor on the federal or national level.

### 2.2 Funding policy for public health service digitization

Despite the missing consensus on public health service digitization, appears the public procurement system as another drawback in Germany (10). Public investments in digitization require a maturity model of the current digitalization state as a prerequisite. The maturity model aims at determining the digital maturity of public health departments. Such formalized analysis must be finalized before any ordering process for software solutions can be started. As a matter of fact, the public health departments have simply no professional staff specialized in this basic digital assessment. Moreover, the general municipal IT structures have no deep insights into the requirements of the respective public service departments. As a matter of fact, nowadays municipal public health services already display digital islands embedded within the public administration. Data transfer possibilities on a community-2-community level are simply not yet existing.

Another disadvantage concerns the acquisition of third-party funding. In fact, Germans municipal public health departments financially household with fixed budgets supplied by local governments whereas external funding application was not foreseen. The idea so far was that public service was financed by public assignments and tax money. This tradition is further accompanied by no or solely minor research activities. Thus, municipal public health services and authorities almost have no experience with the acquisition of third-party funding including proposal applications and fund raising.

In 2021 the Federal Ministry of Health (BMG) came up with a formalized application procedure with two funding track options:

1. Calling for funds via coordinated state initiatives
2. Cross-state one-country-for-all (ELFA) initiatives.

Besides the tight time schedule, the local public health departments were simply lost in this allocation funding. Thus, it did not come as a surprise that almost no cross-state funding project was proposed in Germany. The Federal Ministry of Health funding program thus cemented the already existing digital islands in 16 states and in various municipal public health services.

### 2.3 Defining goals before digitizing public health offices

**Figure 1:**
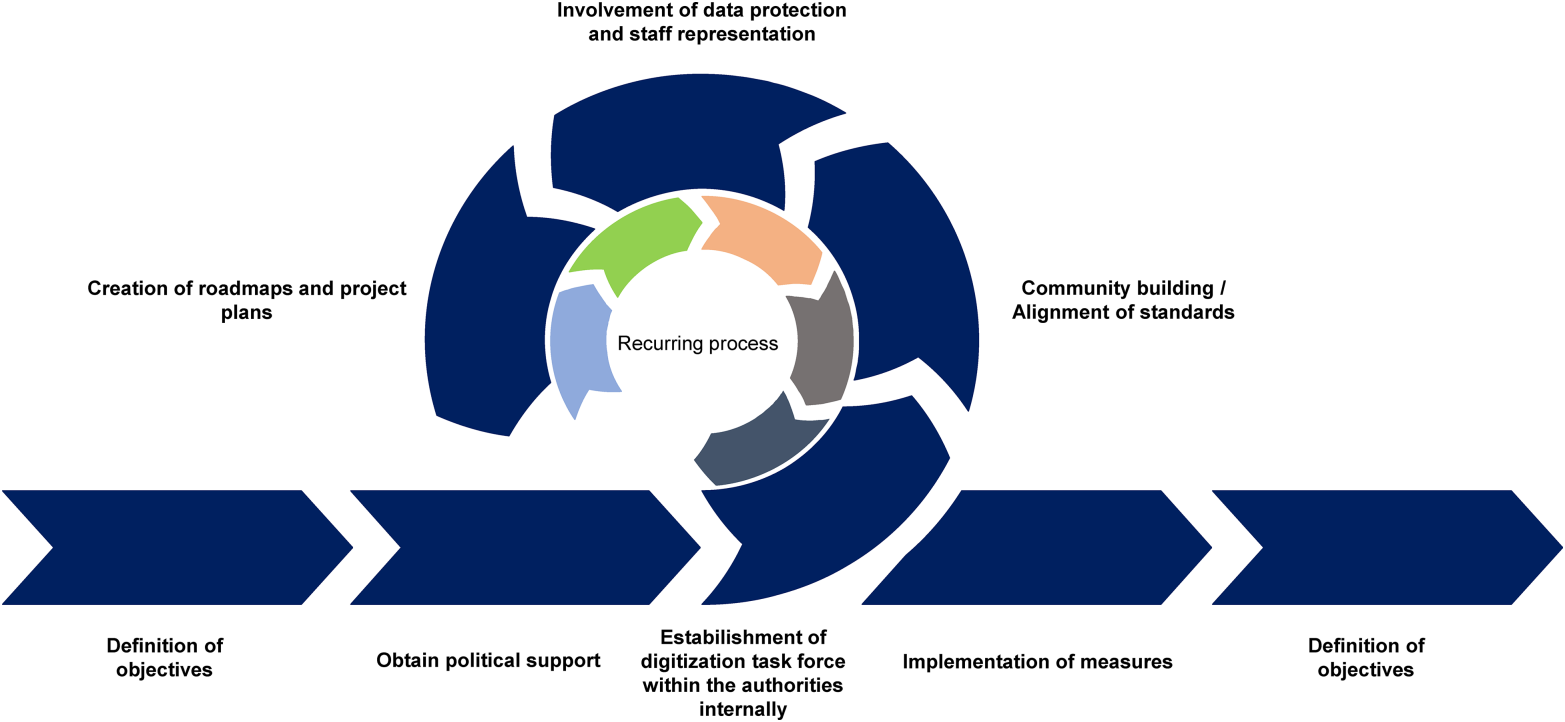
Roadmap of digitalization. The step-by-step implementation of digitalization in the public health service starts with target documentation and runs through the classic milestones of agile process management.

Many public health and administrative institutions are faced with the question how implementation, modernization and digitalization can be done in real world. A classical process path often used in enterprises envisages seven steps (Figure 1). In the first step, the authorities and public offices have exactly to consider the goals they are aiming for in terms of digitization. Additionally, as part of the goal definition is a comparison of the opportunities and risks. Second, opportunities and risks identified should be listed and measures defined to minimize the risks and make the best possible use of the opportunities. The next step is to communicate the goals and ideas to policy decision makers, professionally instruct them and secure financial and conceptual support in the process. Without the assurance of political support, any good idea will simply fail to become reality.

For the operational implementation of the measures, public health authorities should set up their own teams and introduce a so-called “task force digitization” (Figure 1). The team should be representative of the respective office so that all functional needs and requirements can be included in the digitization efforts. In agile processes, the participants should meet in recurring cycles, discuss the upcoming topics and requirements, and distribute tasks. The benefits of such an internal digitization working group would be:

• The digital empowerment of employees
• An implementation of dedicated solutions for their own office
• The identification of employees with the digitization measures
• A stronger orientation towards the user's expertise

**Figure 2:**
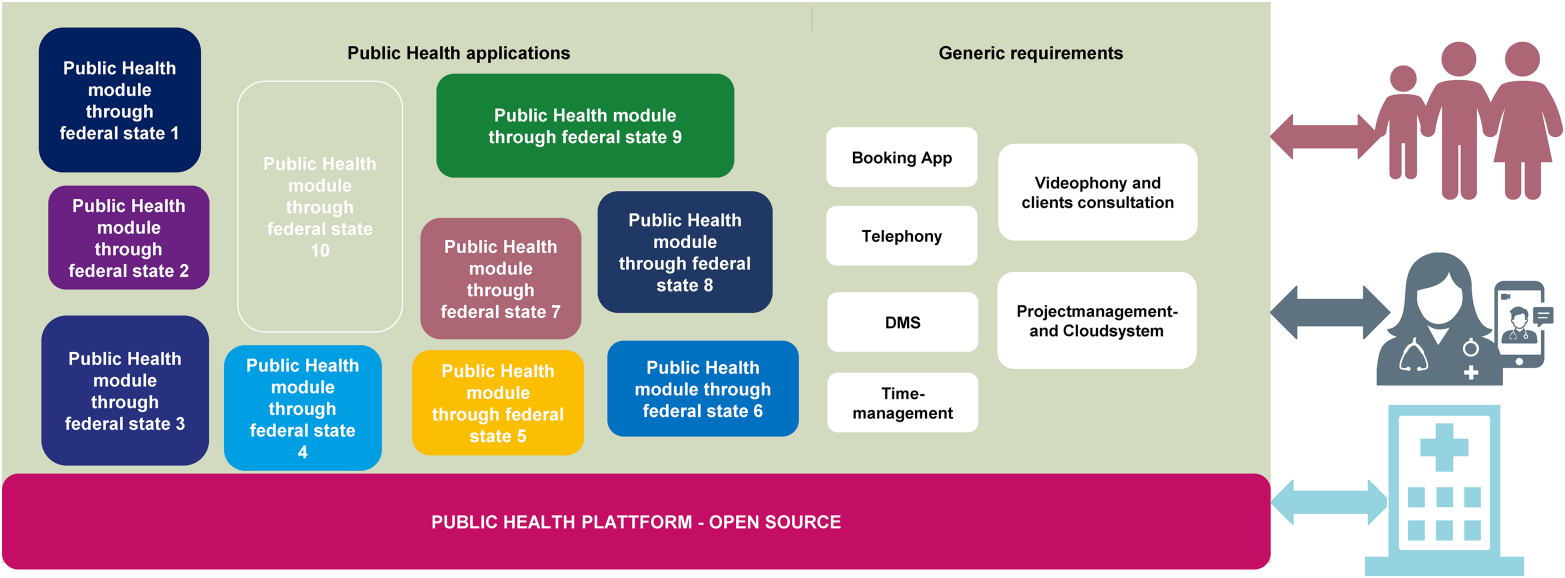
Digitization of the public health service. Division of the digitization of the public health service (ÖGD) into specialist applications (software solutions) and the generic requirements of the public health service. Modular specialist applications (modules as software solutions) can ensure on a common open-source public health base platform, so that those can be implemented barrier-free in the health offices through standardized interfaces. This prevents isolated solutions and technical isolation. A standardized interface can continue enabling communication without media discontinuity from system to system to the other sectors of the public health service (GP practices and hospitals) and to citizens. In addition to technical requirements, generic requirements must also be included in the digital infrastructure so that all channels to citizens and outpatient and inpatient sectors can be multimodally connected and without loss.

Until now, federalism and the lack of exchange among public health agencies have caused taxpayers' money and resources spent multiple times on same solutions. Software applications have been independently developed and without coordination to each other or among them. To avoid these isolated digital island solutions with failing interface connections in the future, it is a prerequisite to increase exchange at the municipal and national level—at best by forming a nation-wide open digitization community. In this context, it is important to agree on an open data and open-source strategy in accordance with the “*public money—public code*” principle (Figure 2).

The next step should be to reach agreements regarding:

• Who coordinates and organizes the community
• Collaborative platforms
• Base architecture and the technology on which to map the digitized modules
• Distribution of tasks and topics
• Common interface standards for integrating the developments on the base platform
• Project plans and roadmaps for overarching projects
• Balance of resources

### 2.4 Involvement of all stakeholders at the very beginning

To overcome data protection issues, data protection officers, staff representatives and the organizational and legal circumstances must be involved early in the planning phase, at best at the beginning. It is important to involve these stakeholders as early as the project planning phase and to keep them involved in the project management process. If possible, the stakeholders should be informed and asked for feedback as early as the preparation of the feasibility study with the included target processes. The previous “*presenting a fait accompli*” approach has not been able to generate any positive experiences in the public health service.

The implementation requires planning dedicated to the needs in one's own office or in one's own municipality or country. This also includes acquiring knowledge in project and process management and to train the employees of public health departments in this. The use of contempory and agile project planning methods should not only be a standard in start-ups and companies of the new economy, but should also find its way into everyday public health work. Preferably, a public authority's own IT coordinator with process and project experience should be permanently installed in the organization. This coordinator should be able to translate the internal needs (requirements) both professionally and technically to the implementation partners, for example programmers or IT service providers. It is important to adjust the salary level of these positions to the conditions of the free market—the current salary level in the public sector is simply too uninteresting for IT and technology-savvy and qualified professionals.

**Figure 3:**
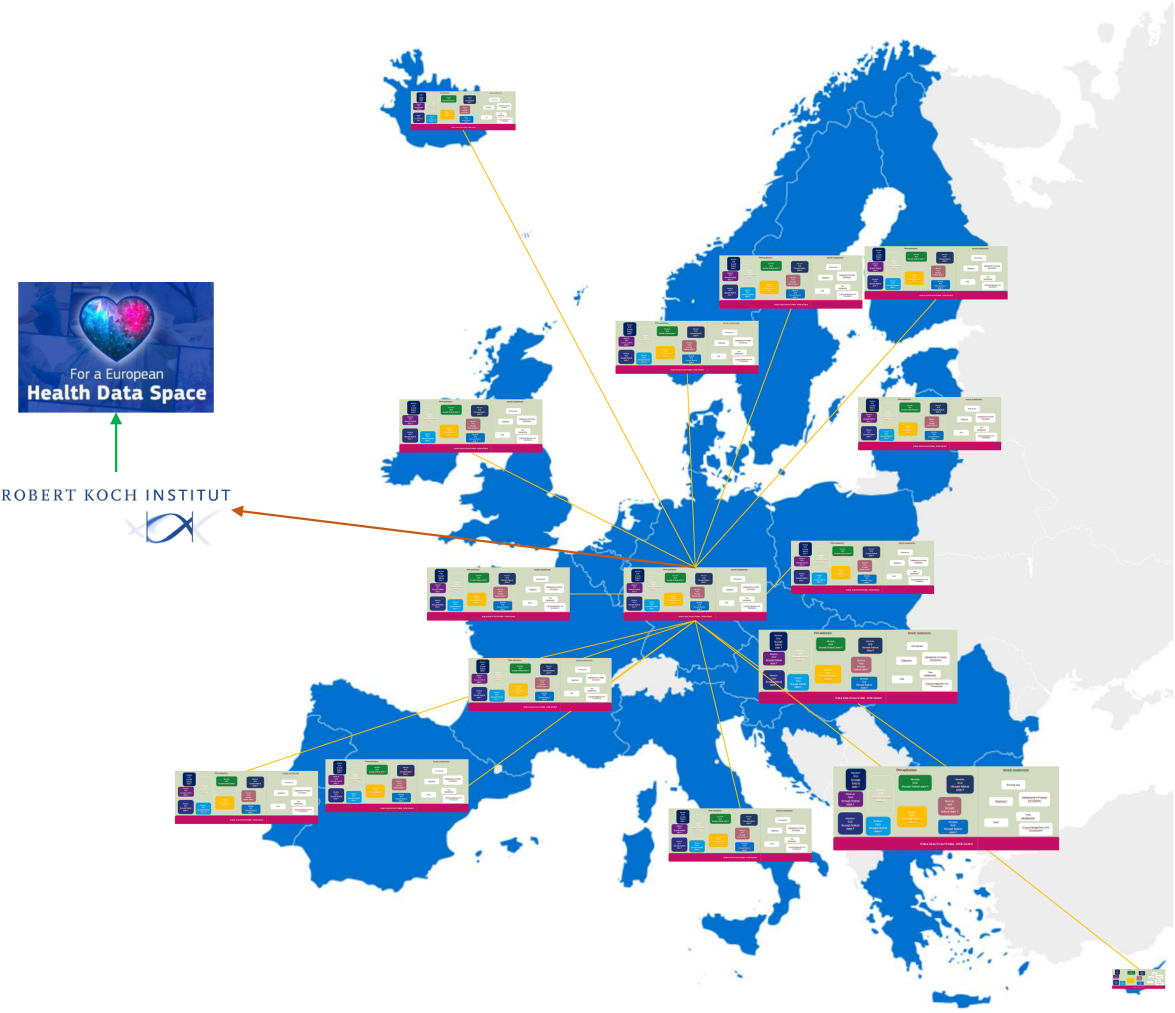
The expansion of a unified public health concept from a German public health perspective. The systems should communicate barrier-free with each other and thus, for example, jointly process and avert infectious incidents. Crises can then be managed jointly in real-time and solutions might be developed much more rapidly. Public health institutions of each country would have to report to its own national public health institute, agency or research institute, which in turn forward and summarize the reports to the European Health Data Space (EHDS) and vice versa.

Based on the project plan for digitalization, the measures can be implemented. Employees should closely communicate with the ctizens, community, stakeholders, data protection officers and service providers to monitor compliance with the project goals and act instantly in the event of any deviations. After successful prototyping and testing by employees, results can be made available to the community for usage, scaling and further development on an open-source platform (Figure 2). The advantage of such concept is that it can also be used European-wide as well as globally. The needs of different countries can also be similar and so, with the template solution, other countries can also apply the system and expand and scale it for their own specific requirements (Figure 3). In this way, it might be possible to reach a common European solution for ongoing and future disease outbreak surveillance and timely prevention. Digital data processing should enable surveillance in real time and barrier-free exchange. Is such a project of this size possible on a cross-national basis? There is good evidence already from commercial enterprises with several cross-border locations world-wide. A good example for such public health endeavor is the open-source software SORMAS (Surveillance, Outbreak Response Management and Analysis System) (11). This open-source software displayed a minimal cost-effective system which can be rapidly scaled up if required. Another reason for the international application of SORMAS was its open interoperability, allowing transparent data exchange. Thus, innovative, and efficient open-source software solutions for public health service can be developed through a European and even global community.

## 3 Discussion

The COVID-19 pandemic revealed a massive disruption of the public health service due to insufficient digitalization, urging now for connectable digital solutions. Investigations of the burden of disease taught us already the need of digital epidemiology tools at the community, national, international, and global scale. Since then, artificial intelligence and data sharing technologies have already been established in clinical medicine (12, 13). Digital epidemiology is thus mandatory for the public health service. Herein, digitization is not restricted to solely communicable diseases and outbreaks but should also include non-communicable diseases surveillance. Moreover, digital epidemiology software must be interoperative to leave the federal niche and can be scaled up globally. Digitalization is a process that is constantly changing according to the technical life cycle. All participants including civil society should be included to shape this agile process. Through intelligent networking, shared objectives and collaborative implementation plans, a digital transformation in the public health service can be modelled on the private sector and create real added value for citizens. This will also lead to stronger public organizational performances and resilience towards the mega challenges such as climate change with subsequent increased zoonosis, migration, and environmental pollution (14).

Informed citizens call for transparent data management and accessibility. Thus, epidemiological data should be made public for tailored community action. Digitalization should be regarded as a human right in a vivid democracy, not just as a technical need. It is now the best time to foster open interface digitization in public health from the national to the European and global level. Sustainable digital solutions need to be interoperable for a global digital surveillance. Ongoing and future communicable disease outbreaks as well as non-communicable diseases need a timely prevention response. From the perspective of citizen digitization must be seen as a human right in an informed, vivid democratic society with increased citizen science (15). Now, after almost overcoming the COVID-19 pandemic it is time to foster digitization in public health service to the global level. Public health service digitization should no longer be the bottleneck for rapid community actions. Participation of all institutions and stakeholders including citizens make sustainable applications possible. A globally connected digitalization makes the public health service future-proof for non-communicable disease surveillance and pandemic prevention, preparedness, and response.
